# Impact of the Simulated Gastric Digestion Methodology on the In Vitro Intestinal Proteolysis and Lipolysis of Emulsion Gels

**DOI:** 10.3390/foods10020321

**Published:** 2021-02-03

**Authors:** Camila Mella, Michelle Quilaqueo, Rommy N. Zúñiga, Elizabeth Troncoso

**Affiliations:** 1 Department of Food Science and Chemical Technology, Universidad de Chile, Santos Dumont 964, Independencia, Santiago 8380494, Chile; c.mellac@utem.cl; 2 Department of Chemistry, Universidad Tecnológica Metropolitana, Las Palmeras 3360, Ñuñoa, Santiago 7800003, Chile; michelle.quilaqueo@amtc.cl; 3 Department of Biotechnology, Universidad Tecnológica Metropolitana, Las Palmeras 3360, Ñuñoa, Santiago 7800003, Chile; rommy.zuniga@utem.cl; 4 Programa Institucional de Fomento a la Investigación, Desarrollo e Innovación, Universidad Tecnológica Metropolitana, Ignacio Valdivieso 2409, San Joaquín, Santiago 8940577, Chile

**Keywords:** emulsion gel, in vitro digestion, gastric motility, gastric emptying, proteolysis, lipolysis

## Abstract

The aim of this work was to study the impact of the methodology of in vitro gastric digestion (i.e., in terms of motility exerted and presence of gastric emptying) and gel structure on the degree of intestinal proteolysis and lipolysis of emulsion gels stabilized by whey protein isolate. Emulsions were prepared at pH 4.0 and 7.0 using two homogenization pressures (500 and 1000 bar) and then the emulsions were gelled by heat treatment. These gels were characterized in terms of texture analysis, and then were subjected to one of the following gastric digestion methods: in vitro mechanical gastric system (IMGS) or in vitro gastric digestion in a stirred beaker (SBg). After gastric digestion, the samples were subjected to in vitro intestinal digestion in a stirred beaker (SBi). Hardness, cohesiveness, and chewiness were significantly higher in gels at pH 7.0. The degree of proteolysis was higher in samples digested by IMGS–SBi (7–21%) than SBg–SBi (3–5%), regardless of the gel’s pH. For SBg–SBi, the degree of proteolysis was not affected by pH, but when operating the IMGS, higher hydrolysis values were obtained for gels at pH 7.0 (15–21%) than pH 4.0 (7–13%). Additionally, the percentage of free fatty acids (%FFA) released was reduced by 47.9% in samples digested in the IMGS–SBi. For the methodology SBg–SBi, the %FFA was not affected by the pH, but in the IMGS, higher values were obtained for gels at pH 4.0 (28–30%) than pH 7.0 (15–19%). Our findings demonstrate the importance of choosing representative methods to simulate food digestion in the human gastrointestinal tract and their subsequent impact on nutrient bioaccessibility.

## 1. Introduction

Nowadays, the main nutritional problems of the world population, such as obesity or undernutrition, could be overcome by the adoption of different technological solutions [1]. These nutritional problems are directly linked to food intake, but it is not only the amount of food consumed that matters. Interactions between proteins, lipids and carbohydrates form the basis of the food matrix facing the digestive system. After digestion in the gastrointestinal tract (GIT), the release of the building blocks of macronutrients (e.g., amino acids, free fatty acids and/or glucose) is dependent on the food matrix structure and the complex processes occurring in the GIT [2]. This explains why, over the years, there has been increasing interest in understanding the behavior of food through the GIT [3].

Food digestion in humans or animals has traditionally been studied with in vivo approaches, but these approaches are expensive, invasive, and have ethical restrictions [3,4]. Consequently, various in vitro digestion systems that attempt to simulate the dynamic, physical and biochemical complexity of the GIT, and particularly systems that mimic the human stomach, have been developed: human gastric simulator (HGS) [5]; TNO gastric small intestinal model (TIM-1) (TNO: Nederlandse Organisatie voor Toegepast Natuurwetenschappelijk Onderzoek) [6,7,8]; dynamic gastric model (DGM) [9]; gastric digestion simulator (GDS) [10]; rope-driven in vitro human stomach model (RD-IV-HSM) [11]. However, some of these systems (HGS, TIM-1, DGM and GDS) have the disadvantage that they do not consider human stomach morphology and, during food digestion, simulated peristaltic movements are not realistically applied. This last point becomes a limitation when the results obtained from these digestion models are interpreted, since the enzyme activity is affected by the mixing of gastric contents and the exposed surface area available for the enzymatic attack, which is itself generated by the disintegration process caused by peristaltic movements. In the case of RD-IV-HSM, although its design considers the human stomach morphology, the gastric contractions generated are produced only at the antral zone. The in vivo contractions that occur throughout the body of the stomach cause the mixing of food with digestive fluids which are then transported by these same peristaltic movements to the small intestine [3,12].

Based on the previous literature, this study focuses on the use of an in vitro mechanical gastric system (IMGS) designed by our research group [13], which has comparative advantages to the systems that are currently available. These advantages are found in the simulation of the peristaltic movements that occur in an adult human stomach (peristaltic frequency and force magnitude), the J-shaped stomach, and reproduction of the gastric pH curve. In this work, the gastric emptying process was incorporated in the operation of the IMGS by applying a controlled pumping process, in order to obtain a greater precision of the digestion kinetics of solid food matrices, such as emulsion gels, as reported by in vivo studies [12]. In particular, emulsion gels are semisolid foods containing emulsified fats and oils [14,15]. In fact, it is known that the distal stomach regulates the rate of gastric emptying of solid meals by coordinated processes involving the antrum, pylorus and duodenum [16]. According to these characteristics, the IMGS can be a useful tool to establish a better understanding of the relationship between the physicochemical properties of foods, its digestion and the subsequent absorption of nutrients. The objective of this work was to study the impact of the methodology of in vitro gastric digestion, analyzed in terms of a system with realistic gastric peristalsis and emptying (IMGS) in comparison with a conventional system based on a stirred beaker operated at constant speed (SB), and gel structure, on the degree of intestinal proteolysis and lipolysis of emulsion gels stabilized by whey protein isolate (WPI). It is important to mention that in this work, measurements of gastric lipolysis of the emulsion gels were not considered because only 5 to 30% of ingested triglycerides are hydrolyzed in the human stomach [17,18,19,20]. Besides, gastric proteolysis was not considered, because a similar degree of gastric protein hydrolysis was found for different whey protein isolate emulsion gels using electrophoresis or titration [21,22], although the degree of gel fragmentation was different between “soft” and “hard” whey protein emulsion gels [21], depending on their structure (fine-stranded or particulate).

## 2. Materials and Methods

### 2.1. Fabrication of Emulsion Gels

Sunflower oil (Natura, Córdoba, Argentina) was purchased from a local supermarket. The major fatty acids of the sunflower oil used in this study were: 6.5% C16:0; 3.9% C18:0; 26.3% C18:1 *w*9 *cis*; 57.3% C18:2 *w*6. Whey protein isolate (WPI) (BIPRO™, Davisco Foods International Inc., Le Sueur, MN, USA) was used as emulsifier and gelling agent. Emulsion gels were prepared from a lipid and an aqueous phase, mixing 30% (*w/w*) lipid phase (sunflower oil) with 70% (*w/w*) aqueous phase (protein dispersion). Protein dispersions were prepared at 9.0% (*w/w*) into phosphate–citrate buffer at pH 4.0 and 7.0, which were subjected to continuous stirring for at least 1 h and at 25 °C. pH 4.0 and 7.0 were chosen because it is widely known that whey proteins form different gel structures (coarse and fine-stranded gels, respectively) at these pHs [23,24,25]. The dispersions were kept overnight at 5 °C to ensure complete hydration of proteins.

Pre-emulsions were elaborated at room temperature by dispersing the lipid phase into the aqueous phase using a rotor–stator homogenizer (Kinematica Polytron^®^, PT 2500 E, Luzern, Switzerland) operated at 6000 rpm for 5 min. The pH of the pre-emulsions was adjusted to 7.0 if required. These pre-emulsions were processed in a high-pressure homogenizer (Gea Niro Soavi, model Panda Plus, Parma, Italy) operating at 500 or 1000 bar and at 3 homogenization steps. Afterwards, the emulsions were subjected to thermal treatment for the formation of the emulsion gels. Emulsions were heated in 50 mL plastic centrifuge tubes in order to set the gels. The tubes were immersed in a water bath (Memmert, model Basic WNB, Schwabach, Germany) at a constant temperature of 90 °C for 30 min. After the heat treatment, samples were cooled to ambient temperature (25 °C) and then were maintained for 24 h at 5 °C for further study.

### 2.2. Characterization of Particle Size of the Emulsions

Mean particle size, particle size distribution and polydispersity index (Pdi) of the emulsions were evaluated using a dynamic laser light scattering instrument (Zetasizer Nano-ZS, Malvern Instruments, Worcestershire, UK). To avoid multiple scattering, emulsions were diluted with phosphate–citrate buffer at a ratio 1:5000 (*v/v*) before measurements. The results were calculated by the instrument software (Zetasizer software, version 6.10).

### 2.3. Textural Characterization of the Emulsion Gels

Cylindrical samples of gels (2.5 cm diameter; 3 cm height) were subjected to texture profile analysis (TPA) and compression test, using a texture analysis machine (Zwick/Roell, model Z0.5, Zwick GmbH & CO, Ulm, Germany). For TPA, gel samples were compressed up to 20% of their original height using an aluminum cylinder probe (5 cm diameter) operated at 0.1 mm/s. After assays, the parameters determined were: hardness (i.e., maximum force during the first cycle of compression), cohesiveness (i.e., related to the internal forces that maintain the structure of a food prior to rupture), and chewiness (i.e., work required to masticate the sample before swallowing) [26,27]. For the compression tests, gel samples were placed between two lubricated plates (5 cm diameter) and compressed at 25 °C at a constant deformation speed of 0.1 mm/s until break [28]. Gel deformation was expressed as the Hencky’s or true strain (*ε_H_*) (Equation (1)) described by:

(1) $$\varepsilon_{H}=-\ln(\frac{H}{H_{0}})$$

where *H* and *H*_0_ (m) are the final and initial height after deformation, respectively. The overall stress acting on the sample during compression was expressed as the true normal stress, which is the normal force to the cylinder cross section divided by the initial area of the sample [28]:

(2) $$\sigma_{t}=\frac{F}{A_{0}}$$

where *σ_t_* is the true normal stress (Pa), *F* is the normal force acting over the gel (N) and *A*_0_ is the cross-sectional area of the gel (m^2^).

### 2.4. In Vitro Digestion Assays of Emulsion Gels

The standardized digestion method proposed by the COST Infogest network [29,30] was used as the basis to perform the in vitro digestion assays, with some modifications. Three phases of in vitro digestion were simulated: oral, gastric and intestinal phase. For gastric digestion, two methodologies were used: (i) IMGS, system composed by a human stomach model and a mechanical system with realistic peristalsis) [13], or (ii) SBg, gastric stirred beaker operated at 150 rpm. The impact of gastric emptying on the in vitro intestinal lipolysis and proteolysis of emulsion gels was studied. After gastric digestion using the IMGS or SBg, the chyme obtained was subjected to intestinal digestion in a double-jacketed glass beaker under continuous stirring (called SBi). The gastric and intestinal digestion times were of 90 and 120 min, respectively.

#### 2.4.1. Preparation of Simulated Digestion Fluids

The preparation of digestive juices was described in our previous works [13,31,32]. The enzymes used here were: α-amylase (porcine pancreas, Sigma-Aldrich, St. Louis, MO, USA), pepsin (porcine gastric mucosa, Sigma-Aldrich, St. Louis, MO, USA), lipase (porcine pancreas, Sigma-Aldrich, St. Louis, MO, USA), pancreatin (porcine pancreas, Sigma-Aldrich, St. Louis, MO, USA), trypsin (porcine pancreas, Fermelo Biotec, Santiago, Chile), and chymotrypsin (bovine pancreas, Fermelo Biotec, Santiago, Chile). Bile extract (porcine, Sigma Aldrich, St. Louis, MO, USA), soy lecithin (Blumos, Santiago, Chile), 1 N HCl (Fisher Chemical, Pittsburg, PA, USA) and 1 N NaOH (Heyn, Santiago, Chile) were also used. Purified water was used for the preparation of all solutions. To simulate the gastrointestinal fluids, simulated salivary fluid (SSF), simulated gastric fluid (SGF) and simulated intestinal fluid (SIF) were prepared, which were adjusted to pH values of 7.0, 3.0 and 7.0, respectively. Simulated fluids were made up of the corresponding electrolyte stock solutions, enzymes, CaCl_2_ and water, according to the standardized digestion method. The concentration of the stock solutions was based on the method proposed by the COST Infogest network [29,30] (Table 1). For all assays, freshly prepared simulated digestion fluids were used.

#### 2.4.2. In Vitro Oral Digestion

In total, 150 g of emulsion gel (~150 mL; pH 4.0 or 7.0) was mixed with 150 mL of SSF (divided into 5 charges, 30 g gel + 30 mL SSF each one), following the mix 1:1 with SSF-containing amylase (75 U/mL) [29,30]. Each charge of this mixture (emulsion gel and SSF) was deposited in dialysis bags. After that, the bags were sealed tightly with adhesive tape, and then were chewed by a human volunteer. As oral residence time depends on the nature and textural characteristics of the material [12], the crushing times were 40 and 60 s for the samples at pH 4.0 and pH 7.0, respectively.

#### 2.4.3. In Vitro Gastric Digestion

**Figure 1:**
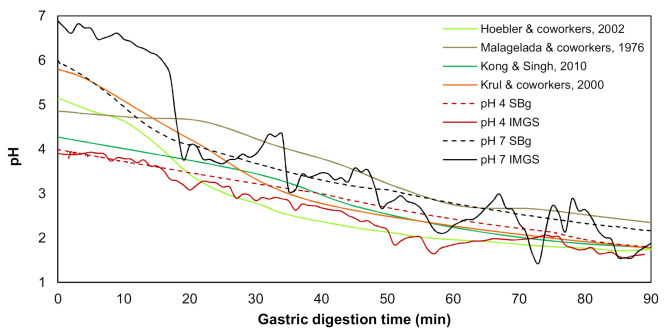
Variation of gastric pH during the in vitro digestion in the IMGS and in a stirred beaker (SBg) of the emulsion gels stabilized by whey protein isolate (WPI), in comparison with the literature data.

As previously mentioned, these assays were carried out in the IMGS or SBg. In both cases, 300 mL of bolus from the in vitro oral digestion was mixed with 300 mL SGF, following the mix 1:1 with SGF-containing pepsin (2000 U/mL) [29,30]. For each methodology (IMGS or SBg) and type of sample (emulsion gel at pH 4.0 and 7.0), a gastric pH curve was performed (Figure 1), since it is known that when a food enters the human stomach, a buffering effect is induced by the food, after which acid secretion produces an exponential decrease in pH. This pH change has been described for in vivo [33,34] and in vitro [5,35] studies, which were used as a reference to build up a programmed pH curve. The pH curve developed for the IMGS considered the gastric-emptying process.

The gastric digestion procedures applied were:

SBg. The bolus was mixed with SGF (37 °C; pH 3.0) and the pH of this mixture was immediately controlled by a pH-Stat automatic titration unit (Metrohm, 902 Titrando, Herisau, Switzerland). In its software (Tiamo 2.4), the parameters were adjusted in order to obtain the expected gastric pH curve by adding defined volumes of 0.5 N HCl solution at different time intervals, until completing the test period (90 min).

IMGS. The bolus was deposited in the simulated stomach of the IMGS, and the SGF was added at a rate of 3.33 mL/min by pumping (Surefusion^TM^, Nipro, Osaka, Japan). The rate of flow of gastric juice was representative of that found in in vivo studies [9,34,36]. Simultaneously, the IMGS operation started, exerting the peristalsis in the stomach at a frequency of 3 cycles/min, physiological value of the human stomach [37,38]. For simulation of the gastric pH curve, the same method used for SBg was applied (Figure 1). After the first 15 min, the gastric emptying valve placed at the pyloric section of the simulated stomach was opened. The gastric emptying was performed intermittently every 10 min using a peristaltic pump (Fisherbrand™ 13-876-2, Fisher Scientific, Suwanee, GA, USA), until completing the whole digestion period (120 min), following the phenomenology reported by in vivo studies [12]. The gastric chyme was transferred at a rate of 10 mL/min to the intestinal phase [36,39], with previous adjustment of pH to 7.0. At the pyloric zone, a membrane (pore size: 2 mm) was incorporated to control the particle size of the chyme passing to intestinal digestion [5]. During IMGS digestion, the overall mechanical force exerted on the emulsion gels samples at pH 4.0 and 7.0 was measured as reported previously [13], obtaining values between the ranges of 0.2–1.2 N and 0.2–1.5 N, respectively. These results are in line with those found in an adult human stomach [37,40].

#### 2.4.4. In Vitro Intestinal Digestion

The intestinal phase was carried out in a double-jacketed beaker at 37 °C subjected to continuous agitation at 150 rpm (SBi). After digesting in the SBg, the gastric chyme obtained (650 mL; pH ~2.0 as shown in Figure 1) was adjusted to pH 7.0 with 1 N NaOH solution. Immediately after that, the chyme was transferred to the SBi and then mixed with 650 mL of SIF (37 °C; pH 7.0), following the mix 1:1 with SIF-containing enzymes [29,30]. For the studies of intestinal proteolysis, the SIF was only composed by trypsin (100 U/mL), pancreatin based on trypsin activity at 100 U/mL, and chymotrypsin (25 U/mL); however, for the intestinal lipolysis assays, the SIF also contained lipase (2000 U/mL) [29,30].

When digesting in the IMGS, the gastric chyme at pH ~2.0 (see Figure 1) was emptied at 10 mL/min into the SBi [36,39], with previous adjustment of pH to 7.0 with 1 N NaOH solution as mentioned previously. The neutralized chyme was then mixed with SIF (37 °C; pH 7.0) pumped (Surefusion^TM^, Nipro, Japan) at a rate of 5.4 mL/min into the SBi, until it reached the total volume (650 mL). The flow rate of the simulated intestinal fluid is in accordance with previous studies reporting values of intestinal secretion in the small intestine ranging from 0.3 to 20.8 mL/min, measured from human volunteers [41,42,43,44].

During intestinal digestion, and after neutralizing the gastric chyme, sample pH was monitored by using an automatic titration unit (Metrohm, 902 Titrando, Herisau, Switzerland) and maintained at a value of 7.0 by adding 0.7 N NaOH solution during the 120 min of digestion. The volume of NaOH solution added to the digested mixture was recorded and used to calculate the intestinal hydrolysis of proteins and lipids.

### 2.5. Quantification of the Intestinal Proteolysis and Lipolysis of Emulsion Gels

#### 2.5.1. Intestinal Proteolysis

After hydrolyzing a peptide bond, one carboxylic group and one α-amino group are produced. During the in vitro digestion at pH 7.0, carboxylic groups release their proton, which can be titrated by NaOH solution using a pH-stat automatic titration [45]. The volume of NaOH solution added can be converted into the degree of intestinal protein hydrolysis (DH), as follows [45,46]:

(3) $$\%\mathrm{DH}=\left[\frac{V_{\mathrm{NaOH}\;\mathrm{proteolysis}}\left(t\right)\times N_{\mathrm{NaOH}}}{{\alpha (\mathrm{RNH}}_{2})\times m_{\mathrm{protein}}\times h_{\mathrm{tot}}}\right]\times 100\%$$

where V_NaOH proteolysis_(t) is the volume of NaOH consumed during a t protein digestion time (L), N_NaOH_ is the NaOH normality (eq/L), m_protein_ is the initial protein mass in the sample (g), h_tot_ is the number of peptide bonds in the protein substrate (8.8 meqv/g for whey proteins [47]), and α(RNH_2_) is the mean degree of dissociation of α amino groups, which can be calculated as follows:

(4) $${\alpha (\mathrm{RNH}}_{2})=\frac{10^{(\mathrm{pH}-\mathrm{pK})}}{\;1+\;10^{(\mathrm{pH}-\mathrm{pK})}}$$

As pK is dependent on the working temperature (37 °C, physiological temperature) and pH (7.0), its value for this study was 7.4, and therefore α(RNH_2_) corresponds to 0.285 [46,48].

#### 2.5.2. Intestinal Lipolysis

Each triacylglycerol (TAG) molecule generates two free fatty acids (FFA) when fully digested. The FFA released from a sample can be calculated from the total amount of TAG present in the original sample, according to [13]:

(5) $$\%\mathrm{FFA}=\frac{{[V}_{\mathrm{NaOH}\;\mathrm{lipolysis}}\left(t\right)-{\;V}_{\mathrm{NaOH}\;\mathrm{proteolysis}}{(t)]\times M}_{\mathrm{NaOH}}\times {\mathrm{MM}}_{\mathrm{lipid}}}{m_{\mathrm{lipid}}\times 2}\times 100\%$$

where V_NaOH lipolysis_(t) is the volume of NaOH solution required to neutralize the FFA produced at lipid digestion time t (L), M_NaOH_ is the molarity of the NaOH solution (mol/L), MM_lipid_ is the molecular mass of the TAG oil (g/mol), and m_lipid_ is the total mass of TAG oil initially present in the sample (g). When lipolysis was analyzed, the final %FFA was obtained by subtracting the respective volume of NaOH solution used in the proteolysis calculation. The times used to estimate the FFA released were the same as those from the degree of intestinal protein hydrolysis. Control runs without enzymes were also performed and subtracted from the reported values.

### 2.6. Statistical Analysis of Data

All experiments were carried out in triplicate using freshly prepared samples. Results are presented as mean values with standard deviations. Analysis of variance was carried out when required using Statgraphic Centurion XVI (version 16.1, Statistical Graphics Corporation, Rockville, MD, USA), including multiple range tests (*p* < 0.05) for separation of the least square means.

## 3. Results and Discussion

### 3.1. Characterization of Emulsions and Emulsion Gels

**Figure 2:**
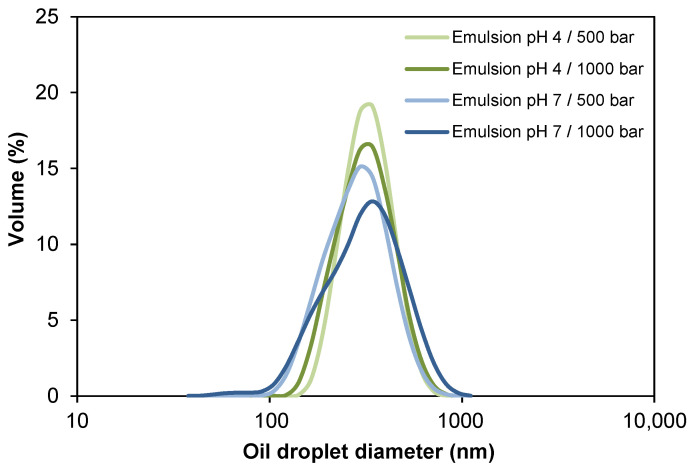
Influence of pH and homogenization pressure on the oil droplet size distribution of O/W emulsions.

The oil droplet sizes and the size distributions for the four oil-in-water (O/W) emulsions stabilized by WPI are presented in Table 2 and Figure 2. Both particle size and Pdi of the emulsions were not significantly affected (*p* > 0.05) by the homogenization pressure, reaching values of 302–328 nm and ~0.19 for emulsions at pH 4.0, and ~275 nm and 0.10–0.17 for emulsions at pH 7.0, respectively. The decrease in oil droplet diameter by increasing the homogenization pressure (500 bar vs. 1000 bar) was only observed in pH 4.0 emulsions (328 nm vs. 302 nm, respectively), whereas in emulsions at pH 7.0, the droplet diameter ranged from 260 nm to 271 nm, respectively. The latter can be attributed to the limited amount of surfactant present to stabilize the droplets formed [49]. It is probable that disruptive forces would be able to generate smaller drops at 1000 bar, but even when there is a sufficient amount of surfactant for emulsion formation, it did not adsorb quickly enough during homogenization. Under turbulent flow conditions, as expected in high-pressure homogenization, newly formed droplets collide, which may lead to rapid recoalescence depending on the extent to which droplets are readily covered by emulsifier molecules [50]. Very short timescales are involved in droplet coverage, and whey proteins were presumably unable to completely stabilize small droplets at 1000 bar.

Unlike the pressure factor, the pH of the emulsions affected the particle size and Pdi, both being significantly lower (*p* < 0.05) at pH 7.0. This can be explained by the lower stability of whey proteins in dispersion at pH 4.0, since when they are in an environment close to its isoelectric point (pH 5.2), they tend to aggregate, giving way to destabilization phenomena (e.g., coalescence or flocculation). Thus, oil droplets would form larger droplets or aggregates in the dispersion [51,52]. All the samples presented Pdi < 0.20 (Table 2), which indicates monodisperse size distributions (Figure 2) [53].

With respect to the textural characterization of the emulsion gels, results from the TPA test (Table 3) indicate that the hardness and cohesiveness were affected only by pH. In the case of hardness, the values were ~10 N for gels at pH 4.0, and ~12 N for gels at pH 7.0. In turn, gels at pH 4.0 and 7.0 presented values of cohesiveness of ~0.45 and 0.87, respectively. Both properties were significantly higher (*p* < 0.05) for pH 7.0 emulsion gels. It is known that fine-stranded WPI gels are formed at pH 7.0, which gives them a more rigid conformation generated by disulfide bonds (covalent interactions) [54,55,56]. This would explain the harder and more cohesive structure of these gels. In addition, it has been indicated that an increase in rigidity of emulsion gels prepared from WPI-stabilized emulsions can be obtained by lowering the emulsion oil droplet size [14]. At pH 4.0, whey proteins tend to aggregate and a coarse (particulate) gel structure with fewer covalent binding points is generated [57], decreasing the hardness and cohesiveness of these gels.

Table 3 shows a congruence between the TPA results and the stress at break values. The pH of the emulsion gels significantly affected (*p* < 0.05) the stress at break, where lower compression stresses (21.7–23.4 kPa) were needed to break the pH 4.0 samples. For pH 7.0 emulsion gels, higher stresses were required (76.2–95.0 kPa) to deform and break up the sample, which can be associated with a longer period of chewing during the in vitro oral digestion (40 s for gels at pH 4.0 vs. 60 s for gels at pH 7.0, as described previously). Accordingly, the chewiness of the emulsion gels was significantly different (*p* < 0.05) when changing the pH from 4.0 to 7.0 and by varying the homogenization pressure. Emulsion gels at pH 7.0 had a higher hardness and were more elastic; hence, a greater force (8.5 N/500 bar vs. 7.4 N/1000 bar) was needed to chew the food and turn it into a bolus, so that there is a proportional relationship between both parameters [58]. The deformation of the samples was significantly affected (*p* < 0.05) only by pH, resulting in a greater deformation before rupture for samples at pH 7.0 due to its structural conformation induced by disulfide bonds, generating stronger and more elastic gels [56].

### 3.2. In Vitro Digestibility of Emulsion Gels

The impact of the type of gastric digestion (IMGS vs. SBg) of emulsion gels elaborated at different pH and homogenization pressures on the in vitro intestinal proteolysis (%DH) and lipolysis (%FFA) was evaluated. pH curves were constructed for both methodologies of gastric digestion to simulate more realistically food digestion in the stomach.

#### 3.2.1. Gastric pH Curves

**Figure 3:**
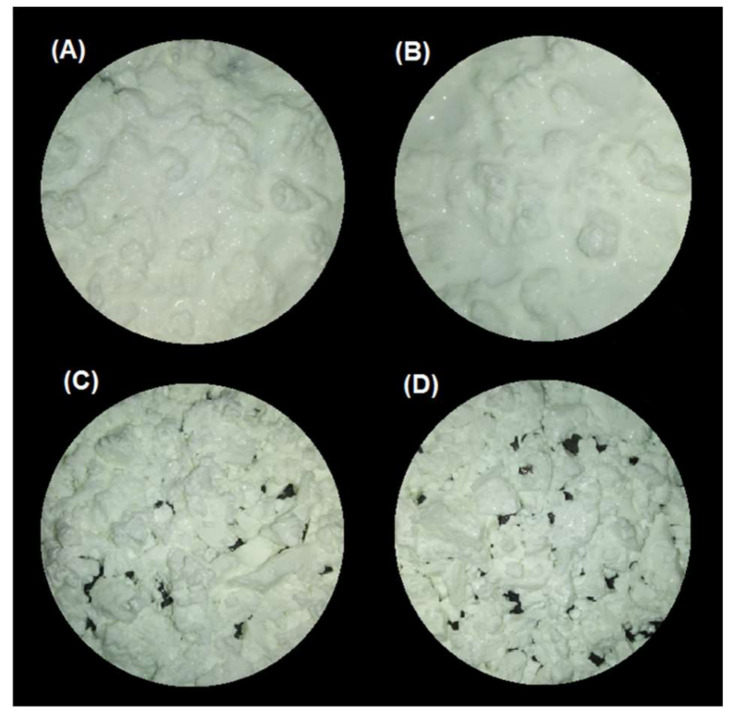
Images of the bolus obtained after oral digestion of the different emulsion gels stabilized by WPI. (**A**) pH 4/500 bar, (**B**) pH 4/1000 bar, (**C**) pH 7/500 bar, and (**D**) pH 7/1000 bar.

The gastric pH curves obtained during digestion of emulsion gels in the IMGS and SBg systems are shown in Figure 1. When beginning the digestion, the gastric pH was ~3.8 and 6.5 for gels fabricated at pH 4.0 and 7.0, respectively. Later, a decrease in pH of the gastric content digested using the IMGS and SBg methodologies was observed. Both gastric digestion systems showed pH changes similar to those reported for in vitro [33,34] and in vivo studies [5,35], with pH values decreasing to ~2.0 after 90 min of digestion. From Figure 1, it is evident that the mixing process in the SBg is faster, because smoother pH curves were obtained in comparison with the noisy pH curves observed when digesting in the IMGS. In the SBg system, continuous agitation was used (similar to a perfectly stirred reactor), which promoted a more homogeneous mixture of the gastric content, independent of gel size resulting from the oral phase by crushing or chewing. In fact, pH 4.0 gels subjected to oral digestion resulted in a paste-like consistency bolus with respect to the particulate state found for gels at pH 7.0 (Figure 3). Regardless of the physical state of the gels after oral digestion, there are no drastic variations in pH during digestion given the effect of the gastric mixing or homogenization. For the IMGS, it should be noted that the pH curves were performed applying realistic peristalsis and gastric emptying. Thus, the mixture of the gastric content (digested gel, SFG and HCl solution) is less homogeneous than in the SBg, which leads to oscillations in the gastric pH curves. These oscillations were less marked for pH 4.0 gels, since the respective bolus formed after oral digestion presented a paste-like consistency (Figure 3), which facilitates the passage of chyme into the intestinal phase (SBi) by action of the gastric emptying process.

#### 3.2.2. Impact of the Type of In Vitro Gastric Digestion of Emulsion Gels on the Degree of Intestinal Proteolysis

**Figure 4:**
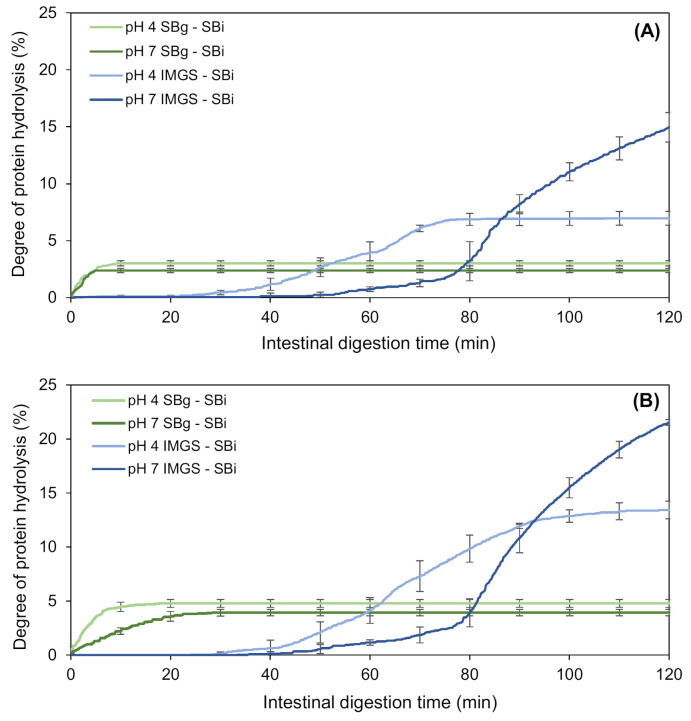
Kinetics of in vitro intestinal proteolysis using the systems IMGS–SBi and SBg–SBi for emulsion gels stabilized by WPI and fabricated at 500 bar (**A**) and 1000 bar (**B**). IMGS: In vitro mechanical gastric system; SBg: in vitro gastric digestion in a stirred beaker; SBi: in vitro intestinal digestion in a stirred beaker.

The kinetics of proteolysis of the emulsion gels during their in vitro intestinal digestion (SBi system) are shown in Figure 4. The nomenclature SBg–SBi refers to the gastric and intestinal digestion assays performed in a stirred beaker, whereas IMGS–SBi refers to the assays of gastric digestion in the IMGS and subsequent intestinal digestion in a stirred beaker. As is clear from Figure 4, the kinetics of intestinal proteolysis of the emulsion gels are markedly different among methodologies. While in the SBg–SBi systems these kinetics are very similar for all samples and do not present a lag phase, the kinetics obtained by the methodology IMGS–SBi showed a difference in shape according to the pH of the studied sample, and showed the existence of a lag phase whose extent was dependent on pH sample.

After gastric digestion, the total chyme obtained is transferred to the intestinal phase, which results in the absence of a lag phase for the samples subjected to digestion SBg–SBi. Accordingly, all the substrate (undigested protein or proteolytic fragments) is available to be hydrolyzed by trypsin and chymotrypsin, and consequently immediate intestinal proteolysis can occur. On the contrary, the lag phase observed for samples assayed with the methodology IMGS–SBi, where the substrate is not hydrolyzed immediately, can be explained by the application of the gastric-emptying process. When the gastric emptying begins, the chyme transported to the intestinal phase is a liquid that contains little substrate available because in the first minutes of gastric digestion, the disintegration of the gels is still reduced. Whether it is hydrolyzed by pepsin or not, the amount of protein that passes to the intestinal phase is low, resulting in the presence of this lag phase. In fact, it has been demonstrated that proteins behave differently at different digestive phases, such as gastric emptying rate [59]. Alternatively, significantly higher percentages of proteolysis (*p* < 0.05) were obtained using IMGS–SBi, with values ranging from ~7.0% to 21.5% with respect to SBg–SBi (Table 4). This may indicate an underestimation of the hydrolysis degrees reported when studying intestinal proteolysis using digestion systems that operate as batch perfectly agitated vessels. From Figure 4, it can be seen that all the samples tested using SBg–SBi quickly increase their digestion rate, but this rate decreases in a short time until reaching a plateau period in which the generated proteolytic products remain constant. Notably, low final extent of proteolysis was found for all samples, with values ranging between 2.4% and 4.8% (Table 4). The rapid decrease in the rate of protein digestion can be caused by the possible inhibition of enzymes due to the effect of the substrate or hydrolytic products accumulated in the system during digestion time [60,61]. Thus, in this study it is possible to infer that the low proteolysis values reached for the SBg–SBi system could be due to trypsin and/or chymotrypsin inhibition during the intestinal digestion of the samples, where the enzymes lose their activity and therefore no reaction products continue to be generated after ~10 min of substrate/enzyme contact. The above is possible because, as already mentioned for this system, after gastric digestion all the chyme is subjected to intestinal digestion. For this reason, the enzyme will be in contact with all the available substrates from the beginning, which induces its possible inhibition.

In a study of enzymatic hydrolysis of lactalbumin, González-Tello et al. [62] demonstrated that there was inhibition of different enzymes by increasing the amount of substrate for the reaction. They concluded that the hydrolysis of whey protein can be explained by an instantaneous and irreversible union of the enzyme with an inhibitor present in the substrate or generated by the instantaneous hydrolysis of some minor component of the protein to be hydrolyzed. This can be corroborated since this phenomenon does not occur in the IMGS–SBi where the substrate is gradually released to the intestinal phase, which is similar to what occurs during an in vivo digestion. Here, we can see how the rate of hydrolytic product release increases according to the amount of substrate that is being transferred and, therefore, the inhibition previously described for the SBg–SBi system is not present when the IMGS–SBi system is used for in vitro digestion assays.

Finally, the type of gastric motility exerted in an in vitro digestion process is fundamental for obtaining values of proteolysis much more representative than using conventional systems (stirred beaker) that may lead to the underestimation of the digestion rate, due to certain phenomena such as the inhibition of proteolytic enzymes.

#### 3.2.3. Influence of the pH of the Emulsion Gels on Intestinal Proteolysis

**Figure 5:**
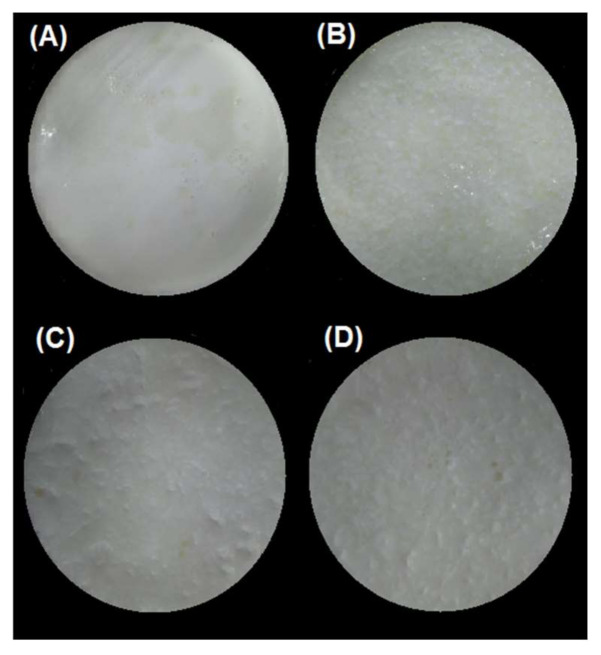
Images of the chyme obtained after in vitro gastric digestion in the SBg system of the different emulsion gels stabilized by WPI. (**A**) pH 4/500 bar, (**B**) pH 4/1000 bar, (**C**) pH 7/500 bar, and (**D**) pH 7/1000 bar.

For the SBg–SBi system, those samples at pH 4.0 showed a significantly higher percentage of hydrolysis (*p* < 0.05) (3.0% and 4.8% at 500 and 1000 bar, respectively) than those at pH 7.0 (2.4%/500 bar and 3.9%/1000 bar) (Table 4). As previously discussed, this can be explained as a consequence of the fact that in these latter samples the gel structure formed is more cohesive and of greater hardness, and therefore after the gastric phase, larger gel particles are still observed (Figure 5). This agrees with the results obtained by Guo and co-workers [21,63], where a higher degree of gel fragmentation was found for “soft” emulsion gels in comparison with “hard” emulsion gels after gastric digestion. In consequence, the attack of hydrolytic enzymes on the protein substrate during the intestinal digestion will be more difficult for gels formed at pH 7.0, resulting in less hydrolysis. Furthermore, possible substrate inhibition can occur as it has already been described.

When analyzing the IMGS–SBi, a lag phase for all samples was observed, with times of 25 and 40 min for gels at pH 4.0 and 7.0, respectively. As in the gastric digestion, the gels at pH 7.0 have a higher time of disintegration induced by peristaltic forces, so the amount of protein that passes into the intestinal digestion at the beginning of gastric emptying is lower and, therefore, the time at which the proteolysis begins is later than in samples at pH 4.0.

**Figure 6:**
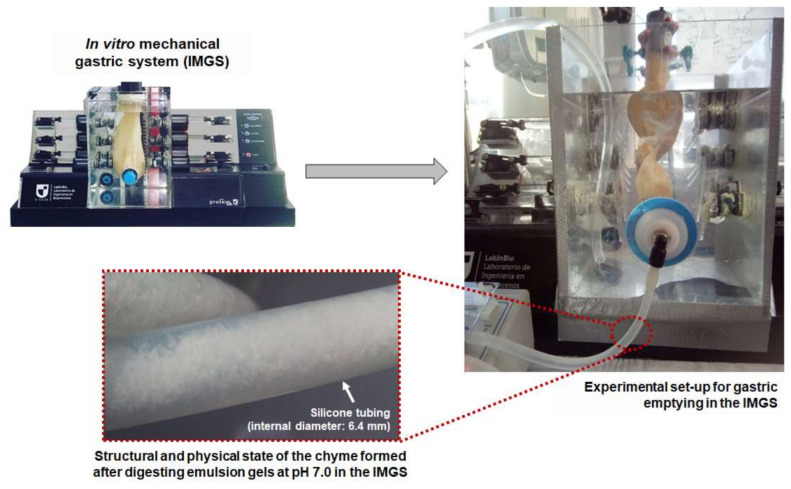
Representative scheme of the gastric emptying process in the IMGS and the chyme formed by digesting emulsion gels at pH 7.0 and emptied into the intestinal vessel for subsequent digestion.

For the IMGS–SBi system, significantly lower percentages of protein hydrolysis (*p* < 0.05) were obtained in the samples at pH 4.0 (7.0% and 13.4% at 500 and 1000 bar, respectively), in comparison with samples at pH 7.0 (15.0%/500 bar and 21.5%/1000 bar) (Table 4). This difference can be attributed to the structure of the emulsion gels at pH 7.0, since compared to smaller aggregates, the larger aggregates provide less cleavage sites for digestive enzymes due to the lower total area, thus showing lower degradation rates [64]. In addition, the tendency of the digestion kinetics differed with the pH of the samples, where at pH 4.0 a plateau phase was reached at the end of the digestion time, but not so in samples at pH 7.0 where the highest values of proteolysis were obtained without reaching a plateau. This is also related to the fact that after gastric digestion, the pH 7.0 samples maintained small fragments of gel that went towards intestinal digestion, during which they disintegrate and release a greater amount of substrate at 80 min. During this time, the rate of digestion increases without reaching a plateau and, therefore, said samples will need more time for a complete digestion. Figure 6 shows a representative image of the structural and physical state of the chyme formed by digesting emulsion gels at pH 7.0 in the IMGS and emptied into the intestinal vessel for subsequent digestion.

#### 3.2.4. Effect of the Type of In Vitro Gastric Digestion of Emulsion Gels on the Intestinal Lipolysis

**Figure 7:**
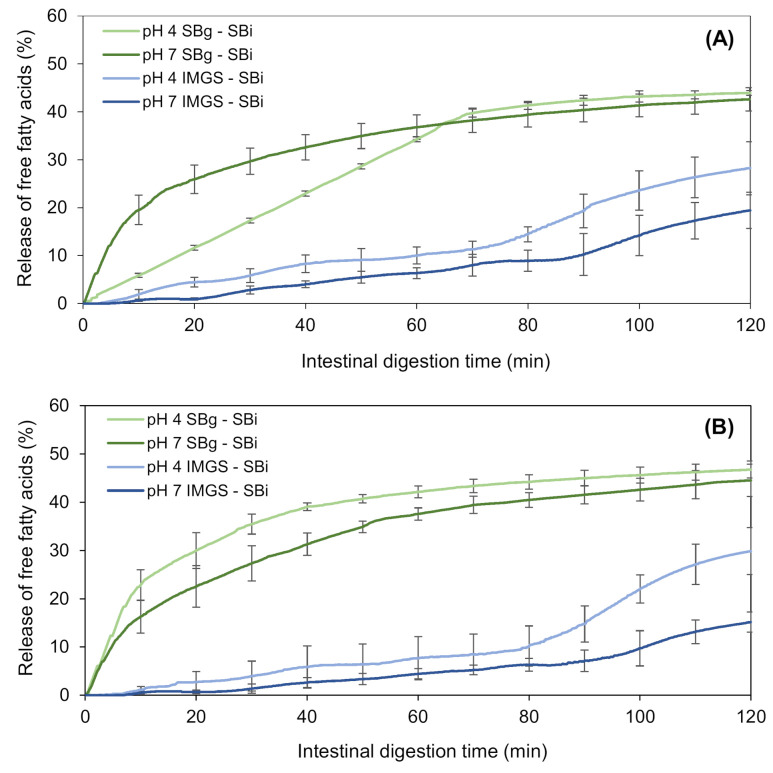
Kinetics of in vitro intestinal lipolysis using the systems IMGS–SBi and SBg–SBi for emulsion gels stabilized by WPI and fabricated at 500 bar (**A**) and 1000 bar (**B**).

The intestinal lipolysis of the emulsion gels was measured by the percentage of free fatty acids (%FFA) released. This percentage was higher for gels digested in the SBg–SBi system than IMGS–SBi, as shown in Figure 7. The final extent of FFA released from gels at 500 bar using SBg–SBi was 43.9% and 42.6% for samples at pH 4.0 and 7.0, respectively, whereas these values were 28.2% and 19.4% when using IMGS–SBi (Table 4). Similar values were obtained for emulsion gels at 1000 bar. These findings reflect the impact that gastric motility and emptying have in food digestion, particularly for solid and semisolid matrices. Here, the type of motility exerted by the IMGS does not induce such a homogeneous chyme, since the peristaltic movements generated in a more realistic way retard the breakdown of emulsion gels (semisolid food matrices) during gastric digestion. This is because, in the human stomach, the maximum destructive force is ~1.9 N; therefore, it is difficult for this one to fragment the food particles with greater hardness into smaller pieces [63]. Then, under these conditions, the size of the pieces of emulsion gel that pass to the intestinal digestion is larger than in the conditions granted by the SBg system, where there is a more homogeneous mixture, so that there is limited access of the enzymes to the surface of oil droplets, decreasing the rate of the release of fatty acids in these samples. Since the rate of lipolysis is controlled by the interfacial area available for the binding of lipase and pancreatin and not by the amount of these [65], lipid digestion can be modulated by designing the structure of the gel around the oil droplets [66].

In addition, the shape of the lipolysis kinetics obtained (Figure 7) agrees with those found for intestinal proteolysis (Figure 4), when comparing both gastric digestion methodologies (SBg vs. IMGS) and subsequent SBi. Thus, when digesting in the IMGS, which involves a mechanism of gastric emptying, the amount of chyme that passes into the intestinal phase is limited, so that the enzymes have less substrate to hydrolyze, resulting in a low rate of FFA release. The latter causes the plateau not to be generated during the 2 h of digestion, requiring a longer time for a complete digestion. This last resembles the kinetics of digestion studied by Barros and co-workers [13], where the lipolysis of WPI-stabilized O/W emulsions was analyzed, which, although it was higher in the IMGS compared to the SBg system, it did not achieve a definitive plateau at the end of the digestion. This has been evidenced by in vivo studies of solid foods (e.g., rice), where at 3 h of digestion, 60% of the dry solids were obtained in the gastric emptying, so a longer time is needed for a gastric digestion of all the content [5]. The same phenomenon was observed in previous in vitro digestion studies of emulsions based on WPI and soybean oil. On the one hand, in the case of emulsions with palm, sunflower and linseed oil, it is only after 200 min of intestinal digestion that a decrease in the rate of fatty acids release is observed [67,68]. Lipolysis kinetics of all samples digested in the SBg–SBi system do not present a lag phase during intestinal digestion. This indicates that an enzymatic attack occurs immediately after starting the intestinal phase. This could be justified by the existence of proteolysis during gastric digestion, where the whey proteins that stabilized the O/W emulsion were exposed to an enzymatic attack by pepsin. Consequently, the stabilizing action of WPI in the emulsion could be altered during gastric digestion, which impacted the subsequent intestinal lipolysis by facilitating the access of lipolytic enzymes to their substrate, attacking them instantaneously.

#### 3.2.5. Influence of pH of Emulsion Gels on Lipid Digestion

The final extent of FFA generated after intestinal digestion in the SBg–SBi system did not show significant differences (*p* > 0.05) with the pH of the gels, reaching a value of ~43.0% FFA (Figure 7; Table 4). When testing in SBg–SBi, a linear rate of lipolysis during the first 65 min of digestion of emulsion gels at pH 4.0 and 500 bar is observed, always with a released %FFA that is lower than the pH 7.0′s gel. After that time, a plateau is reached, which for gels at pH 7.0 this equilibrium stage occurs at 45 min. The result obtained for emulsion gels at pH 4.0/500 bar shows that during gastric digestion, this sample behaved similar to a liquid emulsion (Figure 5), even when it entered the oral phase as an emulsion gel. Therefore, this sample forms a homogeneous and stable matrix, and although there is a greater interfacial area, pepsin action is difficult. This is corroborated with the results of proteolysis for this sample, where less protein hydrolysis was observed. This implies a greater amount of non-hydrolyzed proteins, thus allowing the lipids to be less exposed to an enzymatic attack and also the proteolytic products of the protein to maintain their potential action as a surfactant.

In the case of the kinetics obtained in the IMGS–SBi, it is observed that the four samples under study followed a similar trend during intestinal digestion, presenting a lag phase until approximately 5 min. This is because when gastric emptying in the IMGS begins, the chyme transported to SBi is a liquid with little substrate. Given that during the first minutes the gel disintegration is slow, the available amount of lipids for intestinal hydrolysis is consequently low, resulting in the presence of this lag phase. The digestion trends for these samples did not show significant differences (*p* > 0.05) until the 80 min of digestion, at which time those samples of pH 4.0 began to increase their rate of digestion with respect to the samples of pH 7.0.

The final %FFA obtained in IMGS–SBi is considerably affected by pH, with values of 29.1% and 17.2% for pH 4.0 and 7.0, respectively (Table 4). This difference is due to how the structure of gel was affected by the pH during its elaboration, since at a higher pH, the internal structure of the gel is held together by disulfide bonds, which makes it less susceptible to subsequent mechanical and enzymatic action [54,55]. It is known that in the presence of pepsin, the disintegration rate of a soft gel is higher than a hard gel [63]. Consequently, it can be seen that gels made at pH 7.0 presented values of hardness, cohesiveness and chewiness significantly higher than pH 4.0 gels. Due to these characteristics, during gastric digestion in the IMGS, the disintegration of the structure by the peristaltic action becomes more difficult. As they have a more cohesive internal structure, the enzymatic attack by pepsin is difficult, so that the pieces of gel that finally pass through the pylorus to the intestinal phase are larger. The latter has an impact on intestinal digestion, and although the enzymatic attack of lipase and pancreatin is almost immediate, the rate of fatty acids release is low, achieving a lower final released %FFA than gels at pH 4.0.

Finally, the type of gastric motility and the incorporation of the gastric emptying process during digestion of emulsion gels severely impacts both the kinetics of released FFA and their final extent, which is reflected in the reduction of ~47.9% between the fatty acids released by the IMGS–SBi compared to those obtained by the SBg–SBi system.

## 4. Conclusions

In this work, we studied the impact of the methodology of in vitro gastric digestion and gel structure on the in vitro intestinal proteolysis and lipolysis of emulsion gels. For this, two systems of in vitro gastric digestion were used: a system with realistic gastric peristalsis and emptying (IMGS) and a conventional system based on a stirred beaker operated at constant speed (SBg). After gastric digestion, assays of in vitro intestinal digestion were carried out in a stirred beaker (SBi). Emulsion gels stabilized by WPI were fabricated at different pH (4.0 and 7.0) and homogenization pressures (500 and 1000 bar). The textural characteristics of the gels were determined by the pH of their fabrication, with values of hardness and cohesiveness of ~10 N and 0.45, and 12 N and 0.87 for gels at pH 4.0 and 7.0, respectively. These textural characteristics significantly impacted how the emulsion gels were digested later.

Both intestinal proteolysis and lipolysis of the emulsion gels result in a great difference when using in vitro digestion systems with different operating characteristics. The kinetics of intestinal proteolysis of the gels were markedly different among methodologies. When using the system SBg–SBi, the kinetics were similar for all samples and did not present a lag phase; however, the kinetics obtained by the methodology IMGS–SBi showed a difference in shape according to the pH of the sample, and a lag phase whose extent was dependent on pH sample. In addition, significantly higher percentages of proteolysis (*p* < 0.05) were obtained using IMGS–SBi, with values ranging from ~7.0% to 21.5% with respect to SBg–SBi (~2.4–4.8%). On the other hand, the %FFA released during the intestinal lipolysis of the gels was higher for gels digested in stirred beakers, in comparison with the system IMGS–SBi. The final extent of FFA released from gels at 500 bar using SBg–SBi was 43.9% and 42.6% for samples at pH 4.0 and 7.0, respectively, whereas these values were 28.2% and 19.4% when using IMGS–SBi. Similar values were obtained for emulsion gels at 1000 bar. These findings reflect the impact that gastric motility and emptying have in food digestion, particularly for solid and semisolid matrices.

It is important to understand that the results of in vitro digestion will not only depend on an initial characterization of the food matrix that will be digested (regardless of whether it is in the oral, gastric and intestinal phase), but also on how that matrix disintegrates in each stage of digestion, since the way in which this action occurs in one phase will influence the digestion behavior in the next one. In consequence, the in vitro evaluation of nutrient release should not only be conducted considering the system to be used, but also the structural changes throughout the digestion, thus being able to contribute to the rational design of foods with improved nutritional properties.

## Figures and Tables

**Table 1:** Concentration of stock solutions of simulated digestion fluids.

Constituent	Stock Concentration (mol/L)	Concentration in Simulated Salivary Fluid, SSF (mmol/L)	Concentration in Simulated Gastric Fluid, SGF (mmol/L)	Concentration in Simulated Intestinal Fluid, SIF (mmol/L)
KCl	0.5	15.1	6.9	6.8
KH_2_PO_4_	0.5	3.7	0.9	0.8
NaHCO_3_	1.0	13.6	25.0	85.0
NaCl	2.0	-	47.2	38.4
MgCl_2_(H_2_O)_6_	0.15	0.15	0.12	0.33
(NH_4_)_2_CO_3_	0.5	0.06	0.5	-
HCl	6.0	1.1	15.6	8.4

Data based on [29,30].

**Table 2:** Mean oil droplet diameter and polydispersity index (Pdi) of O/W emulsions stabilized by WPI at different pH and elaborated at different homogenization pressures.

pH	Homogenization Pressure (bar)	Mean Oil Droplet Diameter (nm)	Pdi
4	500	327.8 ± 22.9 ^aA^	0.20 ± 0.04 ^aA^
	1000	301.8 ± 7.1 ^aA^	0.18 ± 0.02 ^aA^
7	500	260.4 ± 21.7 ^bA^	0.10 ± 0.03 ^bA^
	1000	270.8 ± 0.7 ^bA^	0.17 ± 0.01 ^bA^

Different lowercase letters indicate significant differences (*p* < 0.05) between pH of the O/W emulsions, and different capital letters indicate significant differences (*p* < 0.05) between homogenization pressures.

**Table 3:** Textural properties of emulsion gels stabilized by WPI.

pH	Pressure (bar)	Texture Profile Analysis (TPA)			Compression Analysis	
		Hardness (N)	Cohesiveness (Dimensionless)	Chewiness (N)	Stress at Break (kPa)	Strain at Break (Dimensionless)
4	500	10.81 ± 0.55 ^aA^	0.44 ± 0.01 ^aA^	4.57 ± 0.10 ^aA^	21.70 ± 0.83 ^aA^	0.21 ± 0.04 ^aA^
	1000	8.97 ± 0.34 ^aA^	0.46 ± 0.02 ^aA^	3.77 ± 0.53 ^aB^	23.43 ± 1.49 ^aB^	0.24 ± 0.01 ^aA^
7	500	11.31 ± 0.12 ^bA^	0.89 ± 0.02 ^bA^	8.52 ± 0.38 ^bA^	76.21 ± 2.34 ^bA^	0.75 ± 0.12 ^bA^
	1000	13.27 ± 0.28 ^bA^	0.86 ± 0.01 ^bA^	7.40 ± 0.40 ^bB^	94.96 ± 2.51 ^bB^	0.76 ± 0.21 ^bA^

Different lowercase letters indicate significant differences (*p* < 0.05) between pH of the emulsion gels, and different capital letters indicate significant differences (*p* < 0.05) between homogenization pressures.

**Table 4:** Final extent of intestinal lipolysis and proteolysis using different methodologies of in vitro digestion for emulsion gels stabilized by WPI.

Fabrication Conditions of the Emulsion Gels		Methodology of In Vitro Digestion (Gastric–Intestinal)	Final Extent of Intestinal Digestion	
pH	Pressure (bar)		Lipolysis (Free Fatty Acids Released %)	Proteolysis (Protein Hydrolysis %)
4.0	500	SB_g_–SB_i_	43.92 ± 0.48 ^a,A^	3.01 ± 0.22 ^b,A^
	1000		46.74 ± 1.74 ^a,A^	4.78 ± 0.36 ^b,A^
7.0	500		42.59 ± 2.43 ^a,A^	2.39 ± 0.19 ^a,B^
	1000		44.51 ± 3.35 ^a,A^	3.93 ± 0.30 ^a,B^
4.0	500	IMGS–SB_i_	28.24 ± 5.53 ^b,A^	6.97 ± 0.60 ^a,A^
	1000		29.85 ± 4.87 ^b,A^	13.43 ± 0.83 ^a,B^
7.0	500		19.41 ± 3.76 ^a,A^	14.95 ± 1.29 ^b,A^
	1000		15.16 ± 2.09 ^a,A^	21.54 ± 0.26 ^b,B^

Different lowercase letters indicate significant differences (*p* < 0.05) between pH of the emulsion gels, and different capital letters indicate significant differences (*p* < 0.05) between homogenization pressures.

## Data Availability

The data presented in this study are available on request from the corresponding author (Elizabeth Troncoso).
